# Coupling of soil prokaryotic diversity and plant diversity across latitudinal forest ecosystems

**DOI:** 10.1038/srep19561

**Published:** 2016-01-19

**Authors:** Jun-Tao Wang, Yuan-Ming Zheng, Hang-Wei Hu, Jing Li, Li-Mei Zhang, Bao-Dong Chen, Wei-Ping Chen, Ji-Zheng He

**Affiliations:** 1 State Key Laboratory of Urban and Regional Ecology, Research Center for Eco-Environmental Sciences, Chinese Academy of Sciences, Beijing 100085, China; 2 Faculty of Veterinary and Agricultural Sciences, The University of Melbourne, Parkville, Victoria 3010, Australia

## Abstract

The belowground soil prokaryotic community plays a cardinal role in sustaining the stability and functions of forest ecosystems. Yet, the nature of how soil prokaryotic diversity co-varies with aboveground plant diversity along a latitudinal gradient remains elusive. By establishing three hundred 400-m^2^ quadrats from tropical rainforest to boreal forest in a large-scale parallel study on both belowground soil prokaryote and aboveground tree and herb communities, we found that soil prokaryotic diversity couples with the diversity of herbs rather than trees. The diversity of prokaryotes and herbs responds similarly to environmental factors along the latitudinal gradient. These findings revealed that herbs provide a good predictor of belowground biodiversity in forest ecosystems, and provide new perspective on the aboveground and belowground interactions in forest ecosystems.

Belowground prokaryotic communities and aboveground plant communities are tightly associated and form a systematic entity to maintain forest ecosystem functions. They are intimately combined through processes such as biogeochemical nutrient cycling, symbiosis and pathogenicity, *etc*^1^,^2^. Plant diversity regulates gross primary productivity (GPP) with significant consequences on carbon resources and habitat modification for soil prokaryotes^3^, and soil prokaryotic diversity affects plant diversity through mediating nutrient pools and exerting symbiotic and pathogenic influences^4^. Despite that plant and prokaryotic communities are functionally interdependent, it remains largely unknown regarding how soil prokaryotic diversity co-vary with aboveground plant diversity across large spatial scales^5^. Decreasing richness from equatorial to polar areas is the most fundamental pattern of global biodiversity^6^, and it has been proved quite applicable to larger organisms like trees and mammals^1^,^7^. However, whether soil prokaryotes follow the same richness pattern as that of macro organisms remains elusive and little knowledge is available regarding if soil prokaryotic diversity consistently changes with aboveground plant diversity along the latitudinal gradient.

The forest ecosystems cover one third of the terrestrial surface and provide pivotal functions to terrestrial stability and ecological service^8^. Biodiversity in forest ecosystem is experiencing a rapid decline due to the threat from excessive deforestation and global environmental change^9^. Sufficient biodiversity is critically important for the maintenance of ecosystem function^10^,^11^, and studies on large-scale biogeographic patterns of belowground biodiversity could provide a better prediction of forest ecosystem function by linking aboveground plant diversity with belowground prokaryotes^1^,^2^. In forest ecosystems, aboveground plant communities include both trees and understory herbaceous layers. Trees are the most abundant components and the primary producers that generate the highest GPP through photosynthesis in forests. They provide the majority of the organic material inputs for soil through root exudates and litter. Understory herbs, on the other hand, are usually taken as subordinates for their low biomass. Considering the intimate interdependence between producers and decomposers^12^, we hypothesized that soil prokaryotic diversity might co-vary with tree diversity in forest ecosystems.

To test this hypothesis, we investigated the richness (alpha diversity) and beta diversity of belowground prokaryotes and aboveground plants by carrying out a large-scale parallel survey on soil prokaryote, herb and tree communities in 300 quadrats (20 m × 20 m) from tropical rainforest to boreal forest ecosystems in Eastern China (Fig. S1, Table S1). Using both plant community inventory approach and high-throughput sequencing on 16S rRNA gene, we seek to explore how soil prokaryotic diversity couple with tree and herb diversity in forest ecosystems along the latitudinal gradient.

## Results

### Variation of tree, herb and soil prokaryotic richness along latitudinal gradient

**Figure 1:**
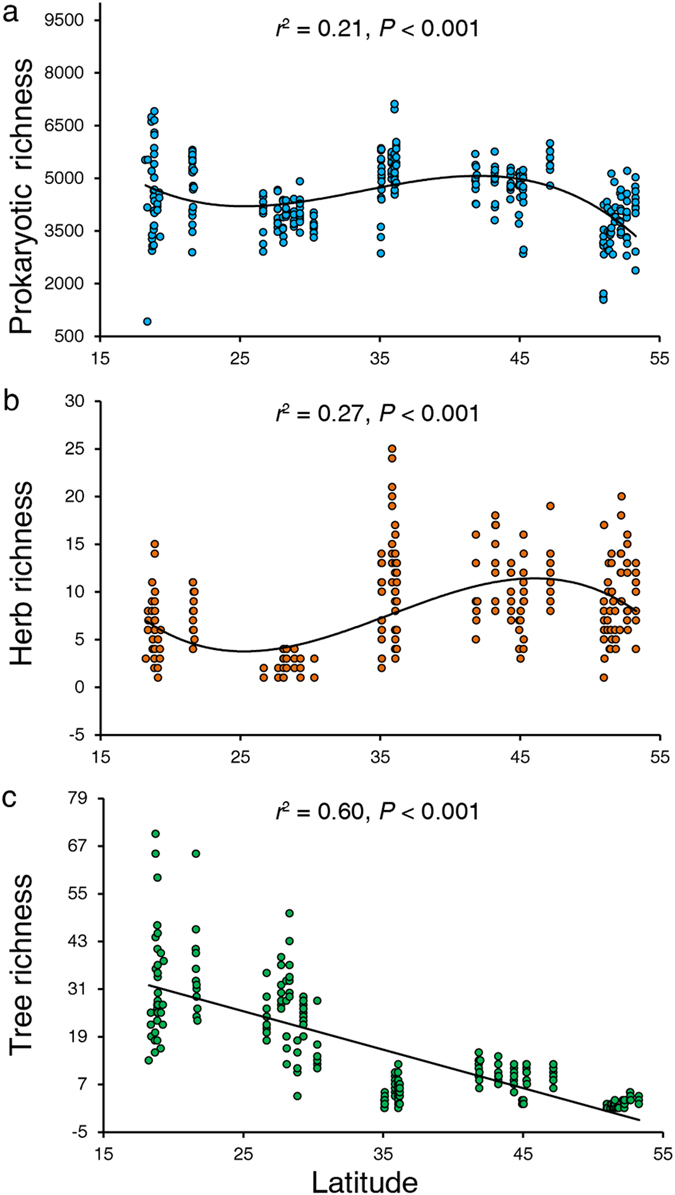
Richness patterns of soil prokaryotes (**a**), herbs (**b**) and trees (**c**) in forest ecosystems across the latitudinal gradient. Prokaryotic richness was demonstrated by the OTU counts defined at the 97% sequence identity at a depth of 45,070 sequences per sample, while herb and tree richness were characterized using their species counts in the quadrats.

We examined the soil prokaryotic alpha diversity patterns by fitting richness with latitude using linear, quadratic and cubic regressions. Contrary to our hypothesis, we found that soil prokaryotes showed a different trend from trees. Soil prokaryotes and herbs decrease in richness in subtropical areas, and linear or quadratic models could not adequately explain the richness patterns of soil prokaryotes or herbs; only a cubic regression could marginally explain their variance (*r*^2^ = 0.21 and *r*^2^ = 0.27 for prokaryotes and herbs, respectively) along the latitude (Fig. 1a,b, Table S2). On the contrary, a linear model could explain the variance in tree richness (*r*^2^ = 0.6) along the latitude, and tree richness exhibited a typical decreasing pattern along the latitudinal gradient (Fig. 1c, Table S2). These results suggested that the latitudinal diversity pattern of soil prokaryotes might be more consistent with herbs rather than trees in forest ecosystems.

### Correlations of soil prokaryotic diversity with tree/herb diversity in forest ecosystems

**Figure 2:**
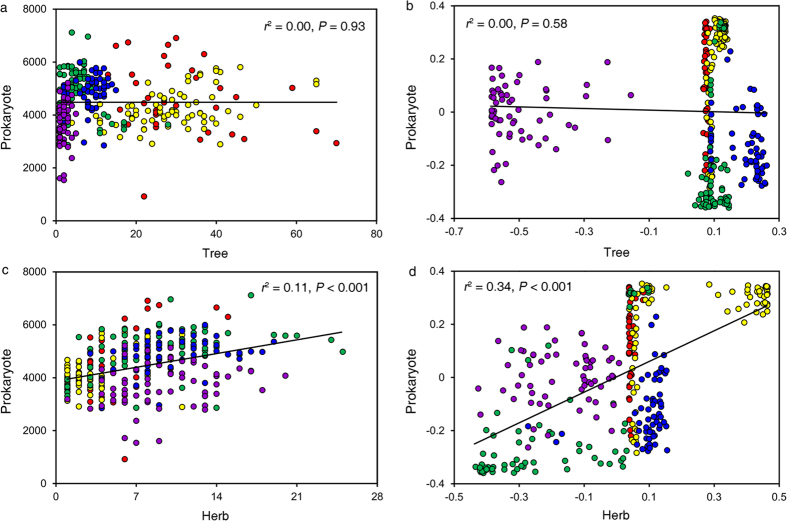
Relationships between tree (**a**,**b**)/herb (**c**,**d**) diversity and soil prokaryotic diversity as determined using Spearman’s correlation coefficient, with x axes showing plant diversity while y axes showing prokaryotic diversity. Both richness (**a**,**c**) and beta diversity (**b**,**d**) indices were employed. Bray-Curtis and unweighted Unifrac distances were used as the measurement of plant and prokaryotic beta diversity, respectively. Correlations of beta diversity were calculated using the first axis of principal coordinates score. Colors of points indicate the latitude (red, <20 °N; yellow, 20 to 30 °N; green, 30 to 40 °N; blue, 40 to 50 °N; purple, >50 °N).

Further correlation analysis revealed that there was no significant (*P* = 0.93) correlation between tree richness and soil prokaryotic richness (Fig. 2a); while a significant (*r*^2^ = 0.11, *P* < 0.001) and positive correlation could be found between herb richness and soil prokaryotic richness (Fig. 2c). The prokaryotic beta diversity was found to be significantly correlated with herbs’ diversity (*r*^2^ = 0.34, *P* < 0.001) but not with trees’ diversity (*P* = 0.58), providing further evidence that soil prokaryotic diversity and herb diversity are more tightly coupled along the latitudinal gradient (Fig. 2b,d). These findings indicate that the community assembly mechanism driving diversity patterns of soil prokaryotes might be similar to herbs but different from trees in forest ecosystems.

### Factors structuring tree, herb and soil prokaryotic diversity along a latitudinal gradient

**Figure 3:**
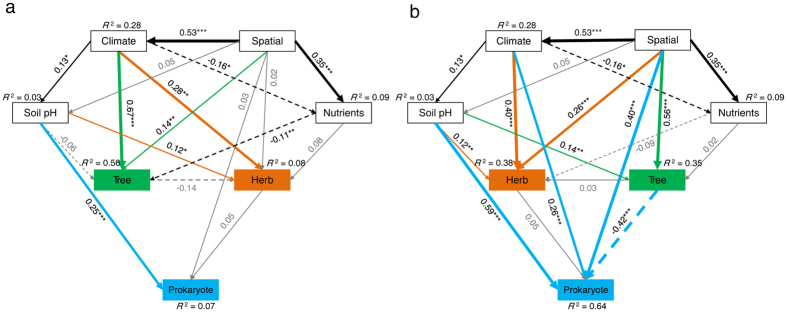
Effects of spatial factors, climate, soil pH and nutrients on alpha (**a**) and beta diversity (**b**) variance of trees, herbs and soil prokaryotes. Nutrients include total nitrogen, soil organic carbon, and available phosphorus. Continuous arrows indicate positive effect while dashed arrows indicate negative effect. The width of the arrows represents the strength of the influence. Goodness-of-fit statistics are evaluated as follows: (Fig. 3a) χ^2^ = 5.4, P = 0.25, d.o.f. = 4, RMSEA = 0.04, AIC = 53.4, GFI = 0.996); (Fig. 3b) χ^2^ = 5.4, P = 0.14, d.o.f. = 3, RMSEA = 0.05, AIC = 55.4, GFI = 0.997. Significant level: *P < 0.05, **P < 0.01, ***P < 0.001. (d.o.f, degrees of freedom; RMSEA, root mean square error of approximation; AIC, Akaike information criterion; GFI, goodness fit index).

We used structure equation models (SEM) to explore how soil prokaryotic diversity was associated with the diversity of herbs and trees under multiple factor regulation. Since alpha and beta diversity are distinct measures of biodiversity^13^, we constructed two separate models for richness and beta diversity based on the currently known mechanism driving latitudinal biodiversity (Fig. S2). Parameters in these models include spatial distance, climatic features (including mean annual temperature and mean annual precipitation), soil nutrients (including soil organic carbon, total nitrogen and available phosphorus) and soil pH. The first model (Fig. 3a) explained 56%, 8% and 7% of the variance in tree, herb and soil prokaryotic richness, respectively. It recognized climate as the dominant driver in determining the latitudinal tree richness pattern. Both climate and soil pH were identified as the significant drivers that directly structure the latitudinal herb richness. By contrast, soil prokaryotic richness was exclusively driven by soil pH, and climate has no significant effect on soil prokaryotic richness. Standardized total effects derived from the SEM revealed that soil pH contributed more to herb and soil prokaryotic richness than to tree richness along the latitudinal gradient (Fig. S4a). The second model explained 35%, 38% and 64% of the variance in tree, herb and soil prokaryotic beta diversity along the latitude, respectively (Fig. 3b). This model revealed that the main driver of tree beta diversity was the spatial isolation, while the climatic condition had little effect. On the contrary, standardized total effect obtained from the SEM revealed that climate impressively regulated herb and soil prokaryotic beta diversity but not on tree beta diversity (Fig. S4b). The effects of spatial isolation were also observed, and soil pH was identified as the predominant factor driving prokaryotic beta diversity. Notably, a significant and negative relationship was observed between trees and prokaryotes after accounting for different environmental factors, indicating a decoupling of tree and prokaryotic beta diversity in forest ecosystems.

## Discussion

There has been extensive evidence that belowground communities vary across different types of vegetation^14^,^15^, but how soil prokaryotic diversity co-varies with aboveground plant diversity along the latitudinal gradient remains less understood. In this study, we found that soil prokaryotes and herbs followed a non-linear richness pattern across five forest ecosystems along the latitudinal gradient. This pattern is not in consistent with current biogeographic patterns of plants or animals, for the latter had much higher richness in tropical areas^16^. Indeed, the latitudinal richness pattern of soil prokaryotes was, to some extent, similar to that of total soil fungi^17^, since richness of both groups was much lower in subtropical areas than in tropical and temperate areas. Similarly to our results, a previous study reported a positive correlation between the beta diversity of soil microbes and herbs in grassland ecosystems^18^. Prokaryotic diversity better coupled with herb diversity than tree diversity under regulation of multiple factors indicated that mechanism driving soil prokaryote and herb diversity pattern might be different from that driving tree diversity along the latitudinal gradient. Traditionally, it is hypothesized that the latitudinal gradient of biodiversity is attributed to the corresponding peak in solar radiation and GPP in equator areas^1^, and climate was considered as the main driver of global plant patterns by Köppen-Geiger^19^. Our result of SEM (Fig. 3, Fig. S4) is consistent with the current perception that the energy and water condition mainly drive the latitudinal plant richness pattern while spatial isolation contributes mostly to plant beta diversity pattern^1^,^20^. Nonetheless, total richness and community composition (beta diversity) of soil prokaryotes are predominantly regulated by soil pH rather than climate along the latitudinal gradient, and herb richness was more sensitive to low soil pH than tree richness. Previous researches usually identified soil pH as the main driver of soil microbial richness patterns across biomes or along the altitudinal gradient^14^,^21^, and our research corroborates that it could have larger impact on microorganism than climate along the latitudinal gradient.

The results that both alpha and beta diversity of soil prokaryotes couples better with herbs rather than trees in forest ecosystems suggest a niche differentiation of trees and herbs in forest ecosystems. Metabolically, herb litters are easier to be decomposed by free-living soil microbes, while the decomposition of tree litters is favored by fungi who are specialized lignin decomposers^22^. In addition, there is an obvious separation in root depth between herbs and trees, since herb roots are denser in surface soil while tree roots could penetrate much deeper^23^. Therefore, prokaryotes in the topsoil are in more contact with herbs. Given the precondition of positive functional association induced similar diversity association between plant and soil microbial communities^18^, our findings indicated that prokaryotic diversity in the subsurface soil habitat might be more consistent with tree diversity in forest ecosystems.

Overall, we provide strong evidence that soil prokaryotic diversity follows similar patterns of those observed for herbs, rather than trees, across large-scale latitudinal forest ecosystems. While trees are considered as the most dominant plant types in forest ecosystems, less dominant herbs can better correlate with the biogeographic patterns of soil prokaryotes. Therefore, our results suggested that herb can be a good predictor for soil prokaryotic diversity not only in grasslands^18^ but also in forest ecosystems. Besides, studies of soil microbial diversity focused mostly on surface soil^18^,^24^, in most cases 0–10 cm of the profile, since the surface layer contains more nutrients and harbors more abundant communities than the subsurface soil in terrestrial ecosystems^25^, but neglected the microbial diversity in deeper horizons. Considering the niche separation of tree (roots) and herb (roots) in forests, it is desirable to examine the relationships between belowground biodiversity and aboveground biodiversity by collecting more representative samples including subsurface soils.

## Methods

### Sites information

This study covers a linear distance of *ca*. 4,000 kilometers across a latitudinal gradient from N18°15′ to N53°18′ in Eastern China (Fig. S1, Table S1). Climatic conditions highly vary along this latitudinal gradient (Fig. S3), with the mean annual temperature (MAT) ranging from −5.6 °C to 25.9 °C and the mean annual precipitation (MAP) ranging from 452 mm to 2,085 mm. Across the 35°3′ latitudinal gradient, we investigated forest bands that ranged from tropic rainforest in the south to subpolar boreal forest in the north. Five major forest types in different climatic regions could be identified, including boreal forest, temperate mixed coniferous forest, temperate deciduous forest, subtropical evergreen forest and tropical rainforest. The sampling sites were selected from the representative mountain ranges in these regions, for example, Mount (Mt) Wuzhi and Xishuangbanna in the tropical region, Mt Tianmu and Mt Wuyi in the subtropical region, Mt Taihang in the warm-temperate region, Mt Xiaoxing’anling and Mt Changbai in the temperate region, and Mt Daxing’anling in the subpolar region. Detailed information about sampling sites and climatic data^26^ could be found in Table S1.

### Vegetation survey and soil sampling

Vegetation survey and soil sampling were conducted from August to September in 2012. Sixty large independent quadrats (20 m × 20 m per each) without known history of anthropogenic disturbance were compartmentalized for community inventory of trees within each forest type, resulting in a total of 300 quadrats for the five types of forests. All the trees with diameter at breast height (DBH) ≥2.5 cm in the quadrats were recorded^27^. Within each large quadrat, three 1 m × 1 m small quadrats were comparted for the inventory of herbaceous plant. Taxonomic information of plants was obtained mainly based on the Angiosperm Phylogeny Group (APG) III system^28^. Assemblages of plant communities were then prepared into a *biom* file^29^ for diversity analysis.

Within each large quadrat, 15 soil cores were randomly taken at a depth of 0–10 cm, mixed and homogenized as one composite sample. A total of 300 soil samples were obtained across all the forest ecosystems (Table S1). Visible stones, roots and other residuals were removed in the field. Fresh soil samples were kept in a freezer before being transported to the laboratory. For each sample, 500 g of fresh soil were air-dried and sieved to 2 mm for physiochemical analyses, and 200 g of fresh soil were freeze-dried and stored under −80 °C for DNA extraction.

### Soil properties analysis

Soil pH was measured with a soil to water ratio of 1:2.5 by a Delta pH-meter. Soil available phosphorus was extracted by 0.5 M sodium bicarbonate solution and determined using a continuous flow analyzer (Skalar Analytical BV, Breda, The Netherlands). Soil moisture content was measured by oven-drying fresh soil samples at 105 °C for 24 h. Air-dried soils were pretreated with H_2_SO_4_ to remove possible carbonate^30^, and analyzed by an Elementar Vario EL III (Elementar Analysen Syetem GmbH, Germany) analyzer to determine soil total nitrogen (TN) and soil organic carbon (SOC).

### High-throughput sequencing of soil prokaryotic communities

Soil genomic DNA was extracted from 0.25 g of frozen-dried soil sample using MO BIO Power Soil DNA Isolation Kits (MO BIO laboratories, Carlsbad, USA), with a slight modification that the initial cell-lysis procedure was performed using a FastPrep-24 DNA isolation system (MP Biomedicals, Santa Ana, USA) at a speed of 5.0 m s^−1^ for 40 s. DNA was extracted three times from each soil sample, and then mixed and homogenized. The quality and concentration of the extracted DNA were assessed using an IMPLEN P-330 NanoPhotometer UV/VIS spectrophotometer (IMPLEN, Munich, Germany).

The primer set 515f/806r^31^ was employed to target the V4 and V5 regions of the prokaryotic 16S rRNA gene, with a 12-bp barcode linked to the reverse primer^32^. The 50 μl PCR reaction mixtures consisted of 25 μl Premix Taq™ (Takara Biotechnology, Dalian, China), 1 μl of each primer (10 μM), 3 μl of template DNA, and 20 μl of sterilized ddH_2_O. The amplification procedure was performed as previously described^32^. The resultant PCR products were purified using the Wizard^®^ SV Gel and PCR Clean-Up System (Promega, San Luis Obispo, USA). The purified amplicons were equimolarly mixed, and 2 × 150 bp paired-end sequencing was carried out on an Illumina Miseq sequencer (Illumina Inc., San Diego, USA).

Raw reads generated from the Miseq paired-end sequencing were merged together using the Fast Length Adjustment of Short reads (FLASH)^33^. A chimera filtering approach UPARSE^34^ was employed as the Operational Taxonomic Unit (OTU) picking strategy at 97% sequence similarity. Representative sequences from individual OTUs generated in UPARSE were processed using the Quantitative Insights into Microbial Ecology (QIIME) pipeline^35^. The resultant OTU map file was converted into a biom file for diversity calculation in QIIME. A resampling procedure was conducted at a depth of 45,070 sequences per sample before diversity calculation.

### Diversity indices

Tree richness was characterized by the species number in the large 400 m^2^ quadrat, while herb richness was characterized by the species number in all the three 1 m^2^ small quadrats inside the large one. Soil prokaryotic richness was characterized by the OTU (97% sequence identity) number at a sequencing depth of 45070 sequences per sample. To estimate the similarity in plant and prokaryotic communities, we generated Bray-Curtis matrices and unweighted Unifrac matrices^36^ for plants and soil prokaryotes, respectively.

### Statistical analyses and modeling

We fitted species richness with latitude using linear and curvilinear (quadratic and cubic) splines to see if soil prokaryotes differ from trees or herbs in the latitudinal richness pattern. We chose the model that could explain a larger part of richness change but with simpler splines as the one that best explained the variance of latitudinal species richness. Analysis of variance (ANOVA) was also used to assess the relationship between latitude and species richness. We examined the relationships between soil prokaryotic richness and tree/herb richness using Spearman’s correlation coefficients. Beta diversity correlation coefficients were calculated using the first axis of the principal coordinates analysis (PCoA) performed in QIIME using the *principal_coordinates.py* script on tree, herb, and soil prokaryotic community variance matrices.

Considering the collinearity of factors across large spatial distances, we used structural equation models (SEM) to determine the relative contributions of different factors on richness and beta diversity variances of trees, herbs and soil prokaryotes along the latitudinal gradient across different forest ecosystems. With knowledge on the current biogeographical framework that climatic and historical factors generally drive the global biodiversity patterns^1^,^14^,^37^, and that edaphic properties intimately determined soil microbial community, we established an *a-priori* model that clarified their effects on tree, herb and soil prokaryotic latitudinal diversity patterns (Fig. S2). Variance inflation factor (VIF) was measured in SPSS 19.0 to judge the colinearity among different factors, and soil pH had strong colinearity with other edaphic factors like SOC (VIF = 7.8) and TN (VIF = 7.7). We separated soil pH from other edaphic factors (including SOC, TN and available phosphorus) because they are usually negatively correlated^21^, and their indiscriminative employment would conceal the contribution of edaphic properties in structuring latitudinal biodiversity. Soil nutrients including carbon, nitrogen and available phosphorus were included in the SEM because they are the base of functional linkage between plant and prokaryotic communities. Mantel test was performed to calculate the interrelationships among those factors aforementioned using *vegan*^38^, and a covariance matrix of these factors was used as the input data for AMOS (Amos Development Co.)^39^. Adequate model fits were determined according to a non-significant χ^2^ test (*P* > 0.05), low Akaike value (AIC), high goodness fit index (GFI) (>0.90), and low root mean square error of approximation (RMSEA) (<0.05)^40^. SEM analyses were conducted using AMOS 17.0.2, and other statistical analyses were performed in R^41^. Standardized total effects of geographic distance, climate (including mean annual temperature and mean annual precipitation), soil pH and nutrient content on plant and prokaryotic diversity were also calculated.

## Additional Information

**Accession code:** The raw data have been deposited with the European Nucleotide Archive database (http://www.ebi.ac.uk/ena) under accession code PRJEB11871.

**How to cite this article**: Wang, J.-T. *et al*. Coupling of soil prokaryotic diversity and plant diversity across latitudinal forest ecosystems. *Sci. Rep*. **5**, 19561; doi: 10.1038/srep19561 (2015).

## Supplementary Material

Supplementary Information
