# Supplementary material for: Coupling of soil prokaryotic diversity and plant diversity across latitudinal forest ecosystems
Source: Sci Rep. 2016 Jan 19;6:19561. doi: 10.1038/srep19561 (PMC4726043; doi:10.1038/srep19561)
Supplement: Supplementary Information [file srep19561-s1.doc]

**Coupling of soil prokaryotic diversity and plant diversity across latitudinal forest ecosystems**

**Authors:** Jun-Tao Wang^1^, Yuan-Ming Zheng^1^, Hang-Wei Hu^2^, Jing Li^1^, Li-Mei Zhang^1^, Bao-Dong Chen^1^, Wei-Ping Chen^1^, Ji-Zheng He^1, 2^*

**Supplementary Materials**

**Fig. S1.** Sampling locations as shown in a map of China. Map was generated using DIVA-GIS 7.5.0.0 software (URL www.diva-gis.org/).

**
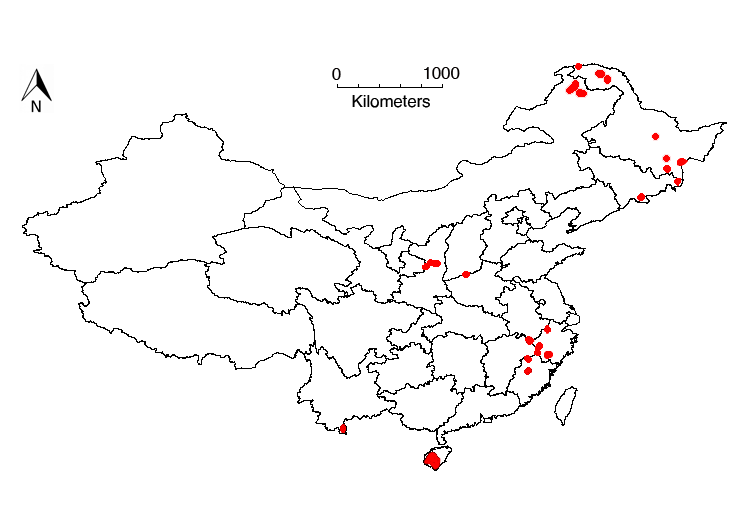
**

**Fig. S2.** A conceptual model for clarifying effects of spatial, climate, soil pH and nutrients in structuring the diversity of trees, herbs and soil prokaryotic communities.

**
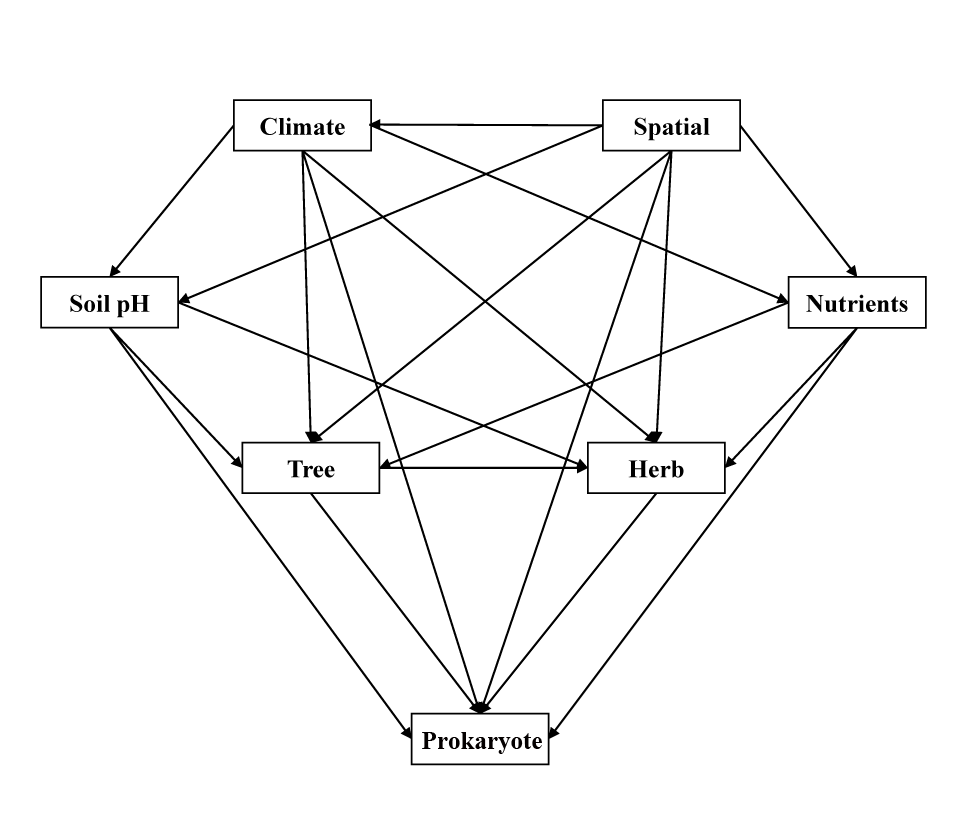
**

**Fig. S3.** Mean Annual Temperature (a) and Mean Annual Precipitation (b) at the quadrats across the study sites along latitude.


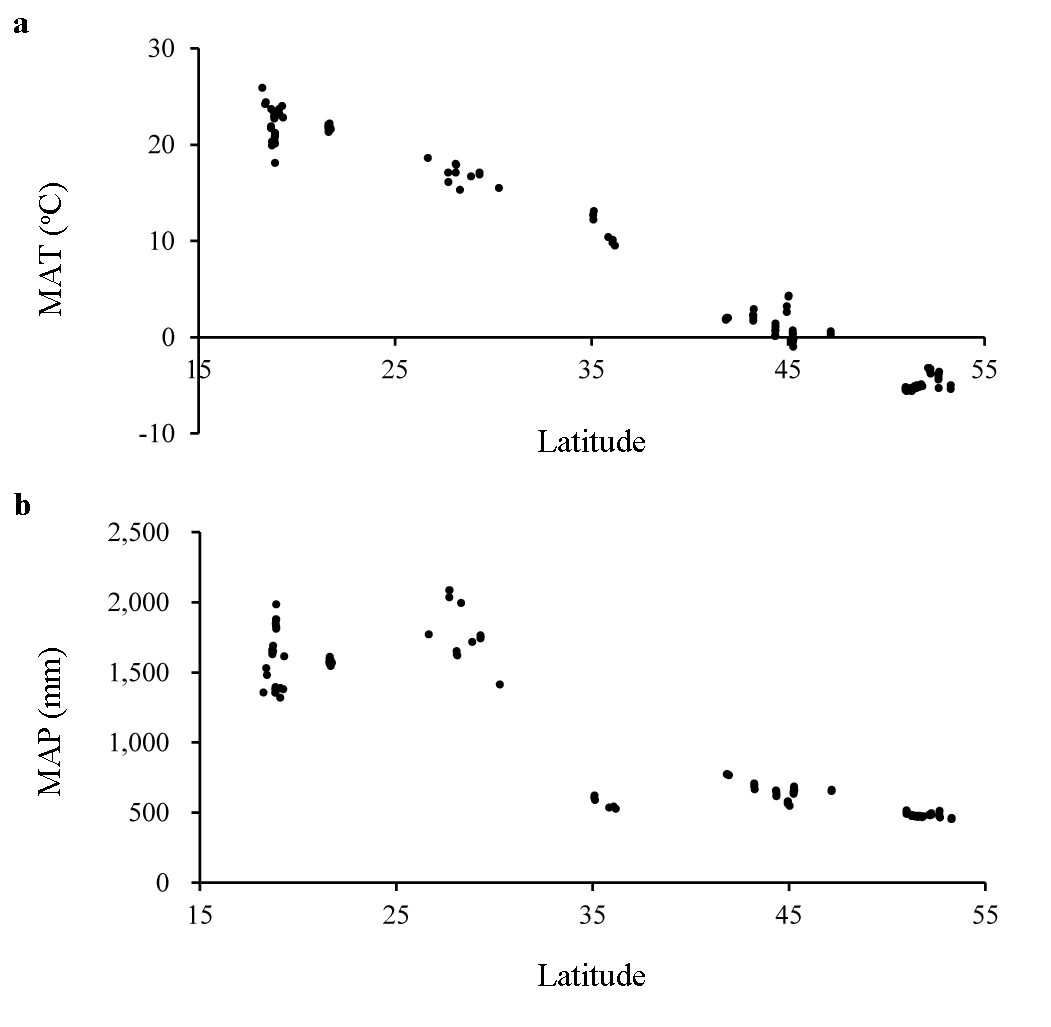


**Fig. S4.** Total effect of factors on latitudinal richness (a) and beta diversity (b) patterns derived from the standardized structural equation model. The standardized total effect clarifies the relative contribution of each factor on diversity variance of tree, herb and soil prokaryote.


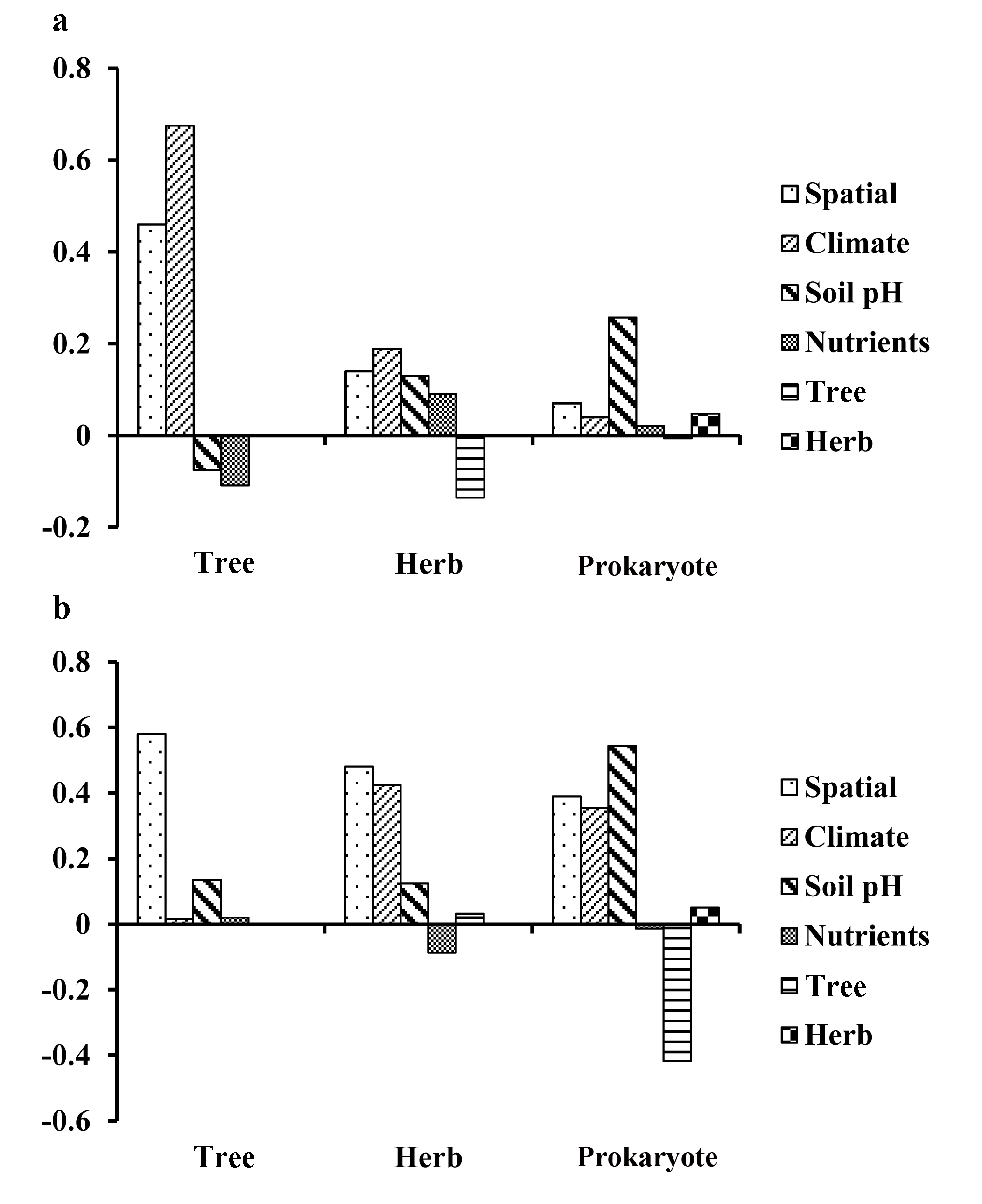


**Table S1.** Characteristics of sites investigated in this study. Data shown were the mean values of replicates in each site. Abbreviations: MAT = mean annual temperature; MAP = mean annual precipitation.

| Site ID | Number of quadrats | Location | Lat(ºN) | Long(ºE) | MAT  (ºC) | MAP  (mm) |
| --- | --- | --- | --- | --- | --- | --- |
| A.JGLC | 10 | Heilongjiang | 53.3 | 122.1 | -5.1 | 454 |
| A.PGZ | 10 | Heilongjiang | 52.7 | 124.0 | -4.1 | 478 |
| A.TEG | 10 | Heilongjiang | 52.2 | 124.7 | -3.4 | 485 |
| A.ALS | 10 | Inner Mongolia | 51.7 | 121.8 | -5.1 | 471 |
| A.JZH | 10 | Inner Mongolia | 51.4 | 121.5 | -5.3 | 475 |
| A.YAL | 10 | Inner Mongolia | 51.0 | 122.2 | -5.4 | 499 |
| A.LS | 10 | Heilongjiang | 47.2 | 128.9 | 0.6 | 654 |
| A.SDHZ | 10 | Heilongjiang | 45.3 | 129.8 | -0.1 | 658 |
| A.FHS | 10 | Heilongjiang | 45.0 | 131.1 | 3.7 | 557 |
| A.MDF | 10 | Heilongjiang | 44.4 | 129.9 | 0.7 | 638 |
| A.FXZ | 10 | Jilin | 43.2 | 130.8 | 2.2 | 692 |
| A.CBS | 10 | Jilin | 41.9 | 127.6 | 1.9 | 771 |
| C.LS | 10 | Shaanxi | 36.2 | 109.1 | 9.5 | 526 |
| C.FX | 10 | Shaanxi | 36.1 | 109.5 | 10.1 | 542 |
| C.YC | 10 | Shaanxi | 36.1 | 109.8 | 9.8 | 540 |
| C.ZWL | 10 | Shaanxi | 35.9 | 108.8 | 10.4 | 535 |
| C.THS | 10 | Henan | 35.1 | 112.2 | 13.1 | 589 |
| C.WWS | 10 | Henan | 35.1 | 112.3 | 12.4 | 614 |
| D.TMS | 10 | Zhejiang | 30.3 | 119.4 | 15.5 | 1412 |
| D.WY | 10 | Jiangxi | 29.3 | 117.8 | 17.0 | 1754 |
| D.JSSD | 7 | Zhejiang | 28.9 | 118.7 | 16.7 | 1716 |
| D.JSXXL | 7 | Zhejiang | 28.3 | 118.5 | 15.3 | 1994 |
| D.YH | 7 | Zhejiang | 28.1 | 119.5 | 17.6 | 1635 |
| D.WYS | 10 | Fujian | 27.7 | 117.7 | 16.3 | 2075 |
| D.SC | 9 | Fujian | 26.7 | 117.7 | 18.6 | 1770 |
| E.YQ | 6 | Yunnan | 21.7 | 101.6 | 21.9 | 1564 |
| E.MLX | 8 | Yunnan | 21.6 | 101.6 | 22.0 | 1571 |
| E.NBH | 10 | Yunnan | 21.6 | 101.6 | 21.9 | 1576 |
| E.BSXBWL | 6 | Hainan | 19.2 | 109.2 | 23.6 | 1410 |
| E.WZS | 13 | Hainan | 18.9 | 109.7 | 20.3 | 1864 |
| E.JFL | 10 | Hainan | 18.8 | 108.9 | 21.8 | 1498 |
| E.SYWZS | 7 | Hainan | 18.5 | 109.6 | 23.7 | 1545 |

**Table S2. Analysis of variance (ANOVA) and** **results of the regressions on latitude and richness. Applicable fittings for tree, herb and soil prokaryotes were marked in bold, respectively.**

|  | Trees | | | | Herbs | | | | Soil prokaryotes | | | |
| --- | --- | --- | --- | --- | --- | --- | --- | --- | --- | --- | --- | --- |
|  | *R*^2^ | *df* | *F* | *P* | *R*^2^ | *df* | *F* | *P* | *R*^2^ | *df* | *F* | *P* |
| linear | **0.60** | 1 | 449.1 | <0.001 | 0.16 | 1 | 57.2 | <0.001 | 0.02 | 1 | 6.3 | 0.01 |
| quadratic | 0.65 | 2 | 281.1 | <0.001 | 0.16 | 2 | 28.5 | <0.001 | 0.08 | 2 | 14.2 | <0.001 |
| cubic | 0.66 | 3 | 190.5 | <0.001 | **0.27** | 3 | 37.1 | <0.001 | **0.21** | 3 | 27.3 | <0.001 |
